# Use of the PRECEDE-PROCEED Model in Piloting Vaccine Promotion and Infection Self-Protection: Intervention Development and Effectiveness Examination

**DOI:** 10.3390/vaccines12090979

**Published:** 2024-08-28

**Authors:** Yao Jie Xie, Longben Tian, Yunyang Deng, Lin Yang, Kin Cheung, Yan Li, Harry Haoxiang Wang, Chun Hao, Gilman Kit Hang Siu, Qingpeng Zhang, Alex Molassiotis, Angela Yee Man Leung

**Affiliations:** 1 School of Nursing, Faculty of Health and Social Sciences, The Hong Kong Polytechnic University, Hong Kong SAR, Chinayan-nursing.li@polyu.edu.hk (Y.L.); angela.ym.leung@polyu.edu.hk (A.Y.M.L.); 2 Research Centre for Chinese Medicine Innovation, The Hong Kong Polytechnic University, Hong Kong SAR, China; 3 Department of Medical Epidemiology and Biostatistics, Karolinska Institutet, 171 77 Stockholm, Sweden; 4 School of Public Health, Sun Yat-sen University, Guangzhou 510080, China; wanghx27@mail.sysu.edu.cn (H.H.W.);; 5 College of Medicine and Veterinary Medicine, The University of Edinburgh, Edinburgh EH8 9AG, UK; 6 Department of Health Technology and Informatics, Faculty of Health and Social Sciences, The Hong Kong Polytechnic University, Hong Kong SAR, China; 7 Department of Pharmacology and Pharmacy, LKS Faculty of Medicine, The University of Hong Kong, Hong Kong SAR, China; 8 Musketeers Foundation Institute of Data Science, The University of Hong Kong, Hong Kong SAR, China; 9 Health and Social Care Research Centre, University of Derby, Derby DE22 1GB, UK; 10 Research Institute for Smart Ageing (RISA), The Hong Kong Polytechnic University, Hong Kong SAR, China

**Keywords:** PRECEDE-PROCEED, COVID-19, vaccination, vaccine promotion, self-protective behaviors

## Abstract

Objectives: This study aimed to tailor and pilot a health education program using the PRECEDE-PROCEED model to promote vaccination and enhance self-protective behaviors against COVID-19 in Hong Kong populations. Study design: Quasi-experimental study. Methods: Phases 1–4 of the PRECEDE-PROCEED model were used to identify the needs for COVID-19 prevention. Strategies to address predisposing, reinforcing, and enabling factors in the PRECEDE-PROCEED model were developed, and an intervention package was generated thereafter. A pre–post experimental study was conducted among 50 participants to preliminarily assess the effects of the intervention based on Phases 5 and 8 of the PRECEDE-PROCEED model. Results: The 3-month intervention package contained 16 health education videos, 36 health tips, individual consultations, regular reminders of vaccination, incentive of anti-epidemic packages, and vaccine booking services. By the third month, 33 participants took a new dose of COVID-19 vaccine, and 5 participants withdrew. The vaccination rate for new dose achieved 73.3% (95% CI: 58.06–85.40%). Compared with the Hong Kong population in the same period, our study demonstrated higher increase in vaccination rate (9.97 vs. 1.36 doses per 1000 person-days). The percentage of early testing in personal and family level increased to 86.7% and 84.4%, respectively (both *p* < 0.05). For correct mask wearing and hand washing, the scores increased from a baseline score of 9.1 ± 1.6 and 4.9 ± 1.3 to 9.5 ± 1.0 and 5.3 ± 1.2, respectively (both *p* < 0.05). Conclusions: The application of the PRECEDE-PROCEED model effectively facilitated the stepwise development, implementation, and evaluation of a health education program for improving vaccination rates and fostering self-protective behaviors against infections.

## 1. Introduction

The outbreak of COVID-19 caused global burdens for all continents, posing critical challenges for healthcare systems, social order, and economic development. Countries worldwide adopted a series of prevention and control methods to combat the spread of COVID-19. The introduction of vaccines at the national level and the cultivation of self-protective behaviors at the individual level took central stage in the containment of the COVID-19 pandemic [1,2]. Moreover, the lack of effective treatment available for COVID-19 emphasized the importance of preventive practices [3]. The World Health Organization (WHO) recommended social distancing, hand washing, face masking, and vaccine uptake as effective preventive practices for COVID-19 containment [4,5]. An ecological study, which included 1,908,197 COVID-19 cases from 190 countries, found that the use of any type of preventive practices could decrease COVID-19 transmission and that the combined use of multiple preventive practices resulted in increased effectiveness [6].

Since vaccines became available, countries worldwide have launched mass COVID-19 vaccination drives. The high uptake rates of COVID-19 vaccination provided immunological protection for vaccinated individuals and whole populations. A global survey reported a 79% rate of COVID-19 vaccine acceptance and a 12% rate of COVID-19 vaccine hesitancy among 23,000 respondents in 23 countries over 3 years [7]. A survey conducted during two waves of the COVID-19 epidemic in Hong Kong from February 2020 to September 2020 found that the rates of COVID-19 vaccine acceptance decreased from 44.2% in the first wave (February 2020) to 34.8% in the third wave (September 2020), and the rates of vaccine hesitancy increased from 55.8% to 65.2% between the two waves [8]. How to improve the vaccination rate remained a big challenge in Hong Kong during the COVID-19 pandemic and post-pandemic era.

On the other hand, the introduction of the COVID-19 vaccine could not negate the importance of other preventive practices, such as self-protective behaviors. A study from the United States found that eliminating mandates on the use of masks after reaching a 50% COVID-19 vaccination rate caused several outbreaks of delta variants, which emphasized the importance of the combined use of the COVID-19 vaccine and self-protective behaviors [9]. Health education took advantage of empowering individuals to accumulate health knowledge and adopt prevention practices, which hold the potential to promote COVID-19 vaccination and foster self-protective behavior. Previous studies have shown the effectiveness of educational interventions in the containment of the COVID-19 pandemic and recommended educational interventions as effective strategies for future outbreak responses [10,11]. 

The PRECEDE-PROCEED model is a widely used and thoroughly evaluated theoretical framework to plan, conduct, and evaluate health education and promotion programs [12,13,14]. It provides an evidence-based framework to identify intervention targets in consideration of health needs and health resources, develops intervention programs under the integration of individual characteristics and context factors, and implements intervention programs incorporating organizational and administrative impediments and supports [15,16,17]. The PRECEDE-PROCEED model has been applied to design diverse interventions to address various health issues, such as chronic disease management and health behavior promotion [18,19]. It was also used to systematically assess the determinants of HPV and influenza vaccination [20,21,22,23]. The application of the PRECEDE-PROCEED model in the context of COVID-19 is unique due to the unprecedented nature of the pandemic, the rapid development and deployment of novel mRNA vaccines, and the widespread vaccine hesitancy. The mRNA-based COVID-19 vaccine candidate BNT162b2. (Pfizer BionTec), as one of the most widely and most effective vaccines, both brought enthusiasm and skepticism. While the rapid development of these vaccines showcased the potential of biotech in responding to global health crises, it also contributed to vaccine hesitancy due to concerns about the long-term effects and new technology involved. These factors created a distinct set of challenges and opportunities, making this a novel case for the model’s application in promoting public health measures. Considering high vaccine hesitancy and the urgent need for COVID-19 prevention in the Hong Kong population, we piloted a health education program by using the PRECEDE-PROCEED model as the theoretical framework to develop and implement the intervention package for promoting COVID-19 vaccination and fostering self-protective behaviors in terms of early testing, hand washing, mask wearing, and social distancing. We also performed a quasi-experimental study to preliminarily evaluate the effect of the intervention package.

## 2. Methods

### 2.1. Study Design 

The present study was reported in accordance with the CONSORT 2010 statement extension to randomized pilot and feasibility trials. It was approved by the Human Subjects Ethical Subcommittee of the Hong Kong Polytechnic University (reference number: HSEARS20210809004) and performed in accordance with the Helsinki Declaration. Written informed consent was obtained from each participant after a verbal and written explanation (via an information sheet) of the purpose and procedures of this study. 

This study had the following two parts: (1) tailoring an intervention package with health education contents based on Phases 1–4 of the PRECEDE-PROCEED model and (2) utilizing a quasi-experimental study to test the effect of this health education intervention package on promoting COVID-19 vaccination, increasing early testing and fostering self-protective behaviors based on Phases 5 and 8 of the PRECEDE-PROCEED model. Phases 6 and 7 were examined in the formal study with a larger sample size and thus not reported here.

### 2.2. Part I: Development of the Intervention Package

**Figure 1:**
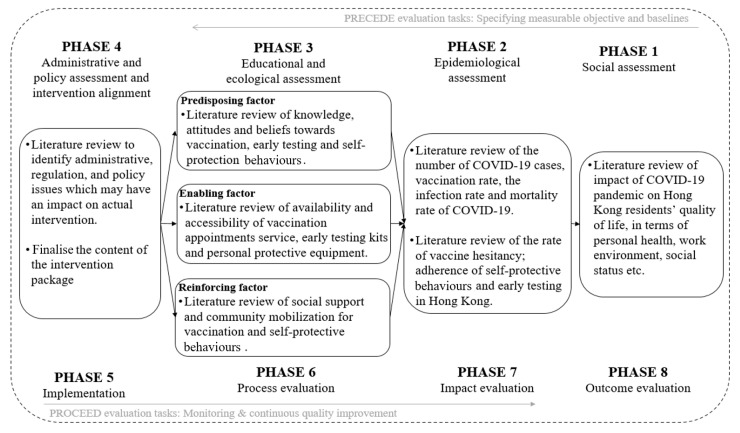
Adapted PRECEDE-PROCEED model and the tasks of Phase 1 to Phase 4 in the context of COVID-19 vaccination promotion and self-protective measures.

We developed an adaptive PRECEDE-PROCEED model under the pandemic context of Hong Kong (Figure 1). The PRECEDE part constituted four assessment phases, which included a social assessment, epidemiological assessment, educational and ecological assessment, administrative and policy assessment, and intervention alignment [15]. 

Phase 1: Social assessment

The social assessment started with a review of the literature on the social influences of Hong Kong residents during the COVID-19 pandemic. Their quality of life and well-being during the pandemic period were assessed [24].

Phase 2: Epidemiological assessment

The epidemiological assessment was conducted through a critical review of the literature and government reports [25,26,27,28]. The number of infections, infection rate, mortality rate of COVID-19 in Hong Kong, vaccination rate and the vaccine hesitancy of COVID-19 in Hong Kong populations, and probability and adherence of self-protective behavior during the pandemic period were all assessed [25,26,27,28].

Phase 3: Educational and ecological assessment

The educational and ecological assessment was performed through a comprehensive review of the relevant literature, which identified the predisposing, reinforcing, and enabling factors of the COVID-19 vaccine and self-protective behaviors [15,25,26,27,28]. Predisposing factors were intellectual and emotional “givens” that made individuals more or less likely to adopt healthy behavior, which included knowledge, attitudes, beliefs, and confidence in the COVID-19 vaccine, early testing, and self-protective behavior [15,25,26,27,28]. Enabling factors were internal and external conditions directly related to issues that help individuals adopt and maintain healthy behavior, which included the availability and accessibility of the COVID-19 vaccine, early testing kits, and personal protective equipment [15,25,26,27,28]. Reinforcing factors followed a behavior, and provided incentives and rewards for the persistence or repetition of the behavior, which included social support and community mobilization for COVID-19 prevention [15,25,26,27,28]. 

Phase 4: Administrative and policy assessment and intervention alignment

The literature review, along with a government report, were used to perform administrative and policy assessment and intervention alignment, which assessed government-disseminated educational materials, government-issued vaccination policies, and government-implemented anti-epidemic policies [29]. An integrative health education package was then developed based on the evidence summarized in Phases 1 to 4.

### 2.3. Part II: Implementation and Evaluation of the Intervention Package

Phases 5: Implementation and Phase 8: Outcome evaluation

According to Phase 5 of the PRECEDE-PROCEED model, a single-arm pre–post experimental study was conducted among 50 participants; then, the effects of the intervention package were evaluated based on Phase 8 of the PRECEDE-PROCEED model [15]. The COVID-19 vaccination rates and self-protective behaviors against COVID-19 were measured as the outcomes at the midterm (1.5 months) and endpoint (3 months) during the 3-month intervention period. The whole experimental study was conducted from February 2022 to May 2022.

#### 2.3.1. Study Subjects and Sample Size Calculation 

A convenience sampling was adopted to enlist 50 individuals residing in noninstitutional settings within Hong Kong. The inclusion criteria were as follows: (1) a resident of Hong Kong, (2) 18 years of age or older, (3) qualified to receive at least one additional dose of COVID-19 vaccine, and (4) willfully consenting to participate in the research by providing written informed consent. The exclusion criteria were as follows: (1) contraindications to the COVID-19 vaccine and (2) an inability to provide vaccination records. 

Based on a previous study, a four-month health education intervention increased the vaccination rate from 35% to 76% [30]. To detect a significant difference with a power of 0.90 and an α of 0.05, we estimated a required sample size of 39 participants for the initial group. Considering an anticipated dropout rate of 15%, the final sample size required was 46 participants.

#### 2.3.2. Recruitment

Participants were recruited through various channels, such as a social network tool (WhatsApp, https://web.whatsapp.com/), the university’s internal email system, the school’s alumni network, and the distribution of fliers and posters in communities and clinics. These posters included details about this study, along with a contact phone number, email address, and QR code. Individuals who were interested in participating could get in touch with the research team for initial registration. Trained research assistants provided interested individuals with information about this study’s objectives and logistical aspects. The initial eligibility of the participants was also assessed. Those who fulfilled the eligible criteria were then invited to participate in this study.

#### 2.3.3. Intervention Implementation

Qualified participants received a 3-month health education program, which was developed based on the evidence from Phase 1 to 4 assessments. The contents of the intervention package and the implementation protocol over the 3-month duration are presented in the Results section as the results for Part 1 of this study. 

#### 2.3.4. Outcome Measures

The primary outcome of the experiment was the COVID-19 vaccination rate, which was calculated by dividing the number of new vaccine doses administered to participants during the intervention period by the total number of participants. To confirm vaccine uptake, we relied on the Hong Kong government’s LeaveHomeSafe software (https://www.fehd.gov.hk/english/licensing/guide_general_reference/COVID19_LeaveHomeSafe.html, accessed on 19 August 2024) or the official vaccine records provided by the participants themselves. The secondary outcomes encompassed early testing, handwashing, mask wearing, and adherence to social distancing. Early testing was determined by asking whether participants or their family members had undergone early rapid testing in the past month, with responses categorized as “Yes” (1) or “No” (0). Handwashing and mask-wearing behaviors were evaluated through a series of 10 and 6 items, respectively, using dichotomous choices (1 for “Yes” and 0 for “No”). These items gauged the correctness of handwashing and mask-wearing practices over the previous month. We then summed the score of each item to create the overall scores ranging from 0 to 10 for handwashing and 0 to 6 for mask wearing, where higher scores indicated more accurate behavior. These items were formulated in accordance with the Department of Health of Hong Kong’s recommendations for proper handwashing and mask wearing [31,32]. Social distancing compliance was assessed by asking participants to rate their adherence to the government’s social distancing regulations over the past month on a 10-point scale, where 0 indicated no adherence and 10 represented the highest adherence. We collected data for all these secondary outcomes at baseline, 1.5 months, and 3 months throughout the study period.

#### 2.3.5. Statistical Analysis

Baseline characteristics, including demographics (sex, age, education, marital status, employment, occupation, living condition, residential area, and income), lifestyle factors (smoking, drinking, and exercise), chronic diseases, and influenza vaccine status, were presented as mean ± standard deviation (SD) for continuous variables and numbers and proportions (%) for categorical variables. Independent *t*-tests and chi-squared tests were employed when appropriate to compare the differences between participants who took the Comirnaty vaccine and those who took the Sinovac vaccine at baseline.

The raw vaccination rates at 1.5 and 3 months were calculated as the number of new vaccine doses taken by the participants during the 1.5-month and 3-month intervention period divided by the total number of participants remaining in the trial at the end of 1.5 and 3 months, respectively. According to the Hong Kong government’s policy on COVID-19 [33], people were not allowed to receive a new vaccine dose within 3 months after COVID-19 infection. Thus, a modified vaccination rate was computed by excluding participants infected with COVID-19 during the intervention period. Subgroup analyses of the vaccination rates were also performed on the basis of the participants’ baseline vaccination dose (first dose vs. second dose).

Meanwhile, given that this was a single-arm quasi-experimental study, we compared the raw vaccination rates of our participants to that of Hong Kong general populations with the same gender (women) and similar age (30–75 years) during the same time period (February 2022 to May 2022) (N_total_ = 2,766,600) [34,35]. The new dose of vaccine per 1000 person-days was calculated and compared between women in our sample and the corresponding Hong Kong population. A scatter plot was established to show the vaccination rates for new doses (per week) in our study and Hong Kong general populations; a loess smooth curve with 95% CI was used to visually demonstrate the rising tendency of the vaccination rate.

Moreover, McNemar’s test was applied to examine the pre–post change in the early testing rates (self and family). A paired *t*-test was used to evaluate the pre–post change in the score of self-protective behavior (hand washing, mask wearing, and social distancing). All statistical analyses were performed using SPSS (version 26.0) and R (version 4.1.3). A two-sided *p* < 0.05 was considered statistically significant.

## 3. Results

### 3.1. Assessment Results Based on Phases 1–4 of the PRECEDE-PROCEED Model 

In accordance with four phases’ assessments, 14 articles or government reports were reviewed to identify the health problems and needs of Hong Kong residents during the COVID-19 pandemic [24,25,26,36,37,38,39,40,41,42,43,44,45,46] (Supplementary Table S1). We also evaluated the decision matrix for each factor in Phase 3 in Supplementary Table S2. In summary, the COVID-19 pandemic significantly impacted the quality of life and well-being of the Hong Kong population with worrisome infection and mortality rates, and insufficient adherence to early testing and self-protective behaviors. Acceptance toward the vaccine and self-protective behaviors was unsatisfactory. Insufficient availability and accessibility toward personal protective equipment coupled with a lack of social support were noticed. Misinformation from unofficial media sources, misunderstanding of official information and health education materials, combined with insufficient explanation efforts by authorities, hampered the public understanding of government policies and strategies against COVID-19.

### 3.2. Structure and Contents of the Intervention Package

The intervention package comprised six components targeting predisposing, reinforcing, and enabling factors. The contents of the intervention package and the implementation protocol are shown in Table 1.

To address predisposing factors, health education materials, including 16 health education videos and 36 health tips, were employed. The videos transformed information from Hong Kong government health education materials about the COVID-19 pandemic, vaccines, rapid testing, and self-protection behaviors into more easily understandable descriptions for residents. These videos were disseminated two to three times per week over the first six weeks and redistributed twice during booster sessions (weeks 7–12). Health tips, which showcased the latest updates on the COVID-19-related situation in Hong Kong, were provided three times per week (Supplementary Table S3).

To address reinforcing factors, individual consultations and reminders of vaccination were instituted. The individual consultations provided one-on-one services to discern and mitigate participants’ hesitancy toward vaccination. The reminders of vaccination encompassed prompts urging participants to get vaccinated. These individual consultations and vaccination reminders alternated on a biweekly basis.

To address enabling factors, anti-epidemic health packages and vaccine booking services were provided. The anti-epidemic health package comprised hand sanitizer, masks, and rapid test kits. The vaccine booking service assisted participants in scheduling appointments through Hong Kong’s online vaccination booking system. 

### 3.3. Effectiveness of the Intervention Package on Vaccination Promotion and Self-Protective Behaviors

#### 3.3.1. Baseline Characteristics of the Participants

**Figure 2:**
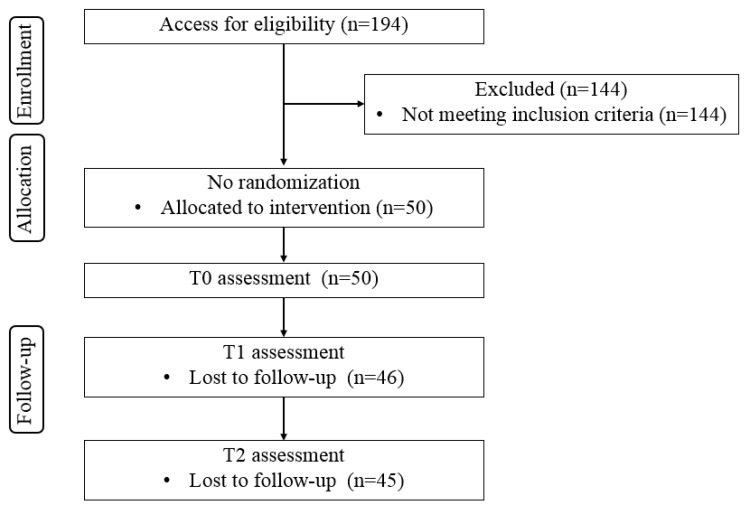
CONSORT flowchart of this study.

As shown in Figure 2, 194 participants were assessed for eligibility at the beginning of the trial. Of these, 144 were excluded for not meeting the inclusion criteria. The remaining 50 participants were allocated to the intervention group and received baseline. The flowchart then shows the progression through this study, with assessments conducted at baseline (T0), after the first follow-up (T1), with 46 participants remaining, and the final follow-up (T2), with 45 participants completing this study.

Table 2 displays the baseline characteristics of the 50 participants. The majority were female (96.0%), with a mean (SD) age of 57.1 (9.3) years. Notably, 68% had at least one chronic disease. Most participants were nonsmokers (86%) and did not consume alcohol regularly (58%). Nearly half did not engage in regular exercise (48%) and were unemployed (48%). A high proportion had education levels of secondary or higher (94%). Marital status showed that 60% were married or cohabiting. Geographically, 54% resided in the New Territories, 32% in Kowloon, and 14% on Hong Kong Island. Income data were disclosed by 74%, with 64.8% reporting a monthly income between 10,000 and 49,999 HKD. Regarding influenza vaccination, 24% had ever received it, and 16% had received it within the past year. 

A total of 48 participants had been administered the first or second dose of either the Sinovac or Comirnaty COVID-19 vaccines before the baseline assessment (Table 2). No significant difference in baseline characteristics was observed between participants who had received the Sinovac or Comirnaty COVID-19 vaccines (all *p* > 0.05).

#### 3.3.2. Vaccination Rate after Intervention

**Figure 3:**
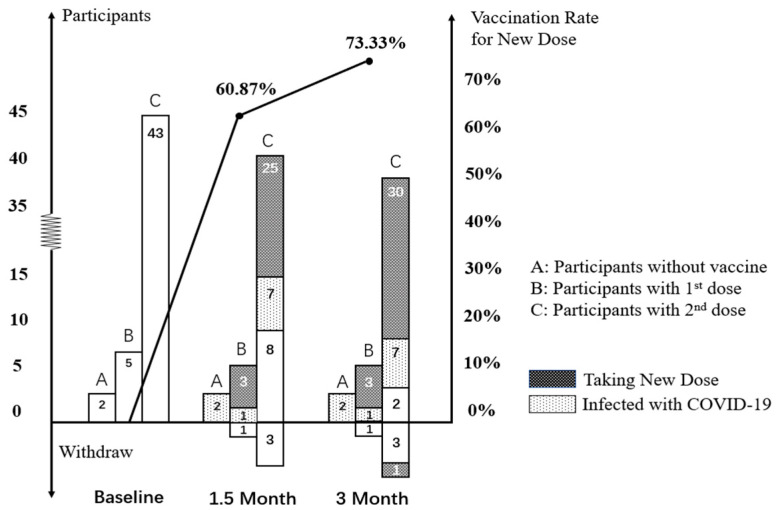
Participants receiving a new dose of COVID-19 vaccine after intervention.

Changes in vaccination rates are shown in Table 3 and Figure 3. Between the baseline and 1.5 months, 28 participants received a new vaccine dose, while 4 participants withdrew. The raw vaccination rate was 60.9% (28 out of 46, 95% CI: 45.4% to 74.9%) at the 1.5-month assessment. During this period, 10 participants were infected with COVID-19, leading to a modified vaccination rate of 73.35% (28 out of 36, 95% CI: 58.1% to 85.4%). Among the 28 participants who received a new dose of the vaccine at 1.5 months, the majority obtained a new third dose (25 out of 28, 89.3%). As illustrated in Figure 3, 43 participants had already received their second dose at the baseline enrollment and were eligible for the third dose during the intervention period. Excluding three participants who withdrew at the 1.5-month point, the raw vaccination rate for the new third dose was 62.5% (25 out of 40, 95% CI: 45.8% to 77.3%). In consideration of the 7 individuals who contracted the virus, the modified vaccination rate for the new third dose was 76.9% (25 out of 33, 95% CI: 60.7% to 88.9%).

Over the course of 3 months, 33 participants received an additional vaccine dose, while 5 participants opted to withdraw from this study, and 10 participants contracted COVID-19. The raw vaccination rates and modified vaccination rates were 73.3% (33 out of 45, 95% CI: 58.1% to 85.4%) and 94.3% (33 out of 35, 95% CI: 80.8% to 99.3%), respectively. Among those who received a new vaccine dose, a substantial majority, i.e., 31 out of 33 (91.2%), received the third dose, with 1 person withdrawing at the 3-month mark. The raw vaccination rates and modified vaccination rates for individuals receiving the third dose of the vaccine were 76.9% (30 out of 39, 95% CI: 60.7% to 88.9%) and 93.8% (30 out of 32, 95%: 79.2% to 99.2%), respectively.

**Figure 4:**
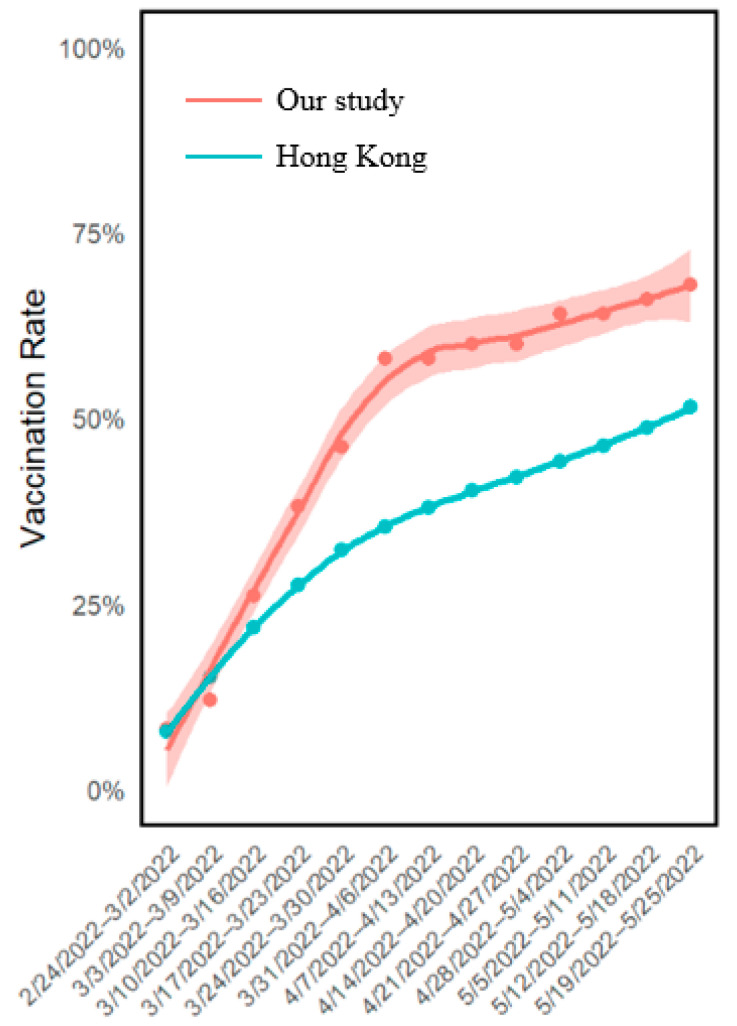
Vaccination rates in our study compared with age- and gender-specific populations in Hong Kong (Feb 2022–May 2022). Figure 4 illustrates the weekly increase in the vaccination rate in women of our study and of Hong Kong general women populations within the same age group (30–75 years) during the same time period (February 2022 to May 2022; *N* total = 2,766,600). A loess smooth curve with 95% CI was used to demonstrate the raising tendency of vaccination rate in our study visually. The upward trend in our study was faster compared to the Hong Kong women general population.

Figure 4 illustrates the weekly increase in the vaccination rate. In our study, the upward trend amounted to 9.97 doses per 1000 person-days among females, surpassing the rate of 1.36 doses per 1000 person-days observed in the same age- and gender-specific (30–75 years, women) Hong Kong population. 

#### 3.3.3. Early Testing and Self-Protection Behavior

Table 4 shows the results of early testing and self-protection behaviors. At baseline, 59.2% of all participants had undergone early rapid testing, and 57.1% reported that their family members had done the same. After the 1.5-month intervention, both of these percentages notably increased to 78.3% (with *p* < 0.05 for both comparisons). After 3 months, these percentages further rose to 86.70% and 84.40%, respectively, again with *p*-values less than 0.05 for both. 

Participants also showed improvements in their self-protective behaviors. The average score (SD) for handwashing behavior saw a slight increase from 9.1 (1.6) to 9.4 (1.3) at 1.5 months (*p* = 0.088), and it significantly climbed to 9.5 (1.0) at 3 months (*p* < 0.05). Similarly, the mean score (SD) for mask-wearing behavior demonstrated improvement, rising from a baseline average of 4.9 (1.3) to 5.2 (1.0) at 1.5 months and 5.3 (1.2) at 3 months (*p* < 0.05 for both comparisons). However, the mean score (SD) for social distancing behavior was 7.6 (1.3) at baseline, significantly increasing to 8.0 (1.1) at 1.5 months (*p* = 0.017) and then slightly decreasing to 7.5 (1.1) at 3 months (*p* = 0.901).

## 4. Discussion

Our study first utilized the PRECEDE-PROCEED model to assess the health needs of Hong Kong residents during the COVID-19 pandemic and develop a health education package. The intervention package significantly improved COVID-19 vaccination rates, surpassing that of the same age- and gender-specific Hong Kong population during the same period. Additionally, the intervention resulted in a significant rise in the proportion of early testing and improvements in correct mask use and handwashing behaviors. Our findings demonstrate the PRECEDE-PROCEED model’s feasibility and efficacy in developing health education programs for promoting vaccination and enhancing self-protective behaviors, offering a practicable framework for future efforts to control infectious disease transmission.

The success of immunization programs highly depends on extensive public vaccination acceptance, making it critical to develop educational programs to increase vaccine uptake during pandemics like COVID-19 [47]. Previous studies have shown varying improvements in vaccination rates with health education. For instance, a pre–post study reported a 32.0% increase in HPV vaccination rate after a 6-month video-based health intervention [48]. A recent meta-analysis of 14 studies found a 39% increase in vaccination rates through community-engaged education [49]. Our study demonstrated a higher increase in vaccination rates than these prior findings, likely due to the multi-component nature of our intervention package and the context of the pandemic. Our intervention included videos, consultations, reminders, incentives, and support services [13]. A meta-analysis found that multi-component health communication campaigns generally lead to greater behavior changes than single-component ones [50]. Furthermore, we implemented targeted educational interventions to tackle the challenges of low vaccination coverage in Hong Kong. At the beginning of our study, the low vaccine coverage observed in Hong Kong was largely due to factors such as vaccine hesitancy and the spread of misinformation. Vaccine hesitancy, particularly during the early stages of the pandemic, was driven by concerns regarding the safety and efficacy of vaccines, which were exacerbated by misinformation circulating on social media and other informal platforms. These interventions were designed to dispel misinformation and deliver personalized communication aimed at addressing the specific concerns of the Hong Kong population. By identifying and addressing the underlying causes of low vaccine uptake, our intervention successfully improved vaccination rates in Hong Kong.

Self-protective behaviors stand as a cornerstone in pandemic control strategies [51]. Our health education intervention significantly enhanced self-protective behaviors during the COVID-19 pandemic. Previous studies also showed improvements following health education. For example, a randomized controlled trial with 18,223 adults found that physician-delivered COVID-19 health messages increased the self-reported protective behavior [52]. A quasi-experimental trial with 219 university students reported increased self-protective behavior scores with a digital-based self-learned educational intervention [11]. Unlike vaccination, which is typically a one-time action, protective behaviors require continuous effort and vigilance, making them more susceptible to changes in public perception and compliance over time. A population-based survey found fluctuating adherence to voluntary protective behavior in Hong Kong populations over two consecutive COVID-19 epidemic waves [53]. The PRECEDE-PROCEED model enabled the development of a holistic intervention tailored to the specific needs within a specific context, helping maintain and enhance protective behaviors over time.

Our pilot study demonstrated a successful example that utilized the PRECEDE-PROCEED model to develop a health education package for vaccination promotion. A study used the first four phases of the model to address predisposing, enabling, and reinforcing factors to COVID-19 vaccination, resulting in 20,792 vaccinations administered at a neighborhood site over 16 weeks [54]. A meta-analysis found that interventions using the PRECEDE-PROCEED model could significantly improve health knowledge [13]. This evidence supports the PRECEDE-PROCEED model as an effective framework for health education. The model’s strengths included its systematic approach to planning and evaluating health education programs, ensuring evidence-based and population-centered interventions [55]. Another key advantage of the PRECEDE-PROCEED model was its flexibility in responding to dynamic changes in community health needs [56]. During a pandemic, this model allowed for continuous assessment and adaptation of strategies, ensuring interventions remain relevant and effective over time. Additionally, its step-by-step methodology facilitated an easy adoption and implementation, even for those unfamiliar with the model, showcasing its potential for future community-based health promotion programs [57].

Several limitations of the present study should be noted. Firstly, the sample size of the quasi-experimental study was relatively small, with only 50 subjects included. Nevertheless, this was just a pilot study to assess the preliminary effects of the health education program against COVID-19 infection. A formal randomized controlled trial with more participants should be conducted to validate further the findings of this pilot study in the future. Secondly, a single-arm design was employed. Since no control group for comparison, it might lead to uncertainty in assessing the intervention effects to some extent. Alternatively, we compared our results with the Hong Kong general populations with the same gender (women) and similar age group (30–75 years) during the same period. This comparison strongly verified the positive effect of the intervention on COVID-19 vaccination rate. Nonetheless, given the data on potential confounders, the results of the quasi-experimental study should be interpreted with caution. 

## 5. Conclusions

The present study tailored and piloted a health education intervention based on the PRECEDE-PROCEED model to improve vaccination rates and enhance self-protective behaviors against COVID-19 in Hong Kong. The PRECEDE-PROCEED model facilitated the development of the intervention package, which allowed the integration of individual and environmental factors and enable the consideration of organizational, administrative, and policy barriers or support. Our study findings support the feasibility and effectiveness of using the PRECEDE-PROCEED model to develop a comprehensive intervention for promoting vaccination and enhancing self-protection behaviors, which provides a referential approach for future studies in controlling the transmission of infectious diseases in the population. 

## Figures and Tables

**Table 1:** Intervention protocol developed from PRECEDE-PROCEED model.

Time	Core Intervention Package			
	**Strategies to Address Predisposing Factors**		Strategies to Address Reinforcing Factors	Strategies to Address Enabling Factors
Week 1	**Health education video 1:** Introduction to the project. **Health education video 2:** Etiology, symptoms, and transmission of COVID-19. **Health education video 3:** Epidemiology of COVID-19.	**Health tips** * Tips 1–3	Individual consultant	**Delivering anti-epidemic package**
Week 2	**Health education video 4:** Reasons for taking COVID-19 vaccines and two types of COVID-19. vaccines in Hong Kong. **Health education video 5:** Effectiveness and safety of Comirnaty and Sinovac. **Health education video 6:** Safety and side effects of COVID-19 vaccine.	Tips 4–6	Reminder of vaccination	**Vaccine booking service**
Week 3	**Health education video 7:** COVID-19 vaccination program in Hong Kong. **Health education video 8:** Vaccine hesitation. **Health education video 9:** Breaking vaccine prejudice.	Tips 7–9	Individual consultant	
Week 4	**Health education video 10:** Arrangement on COVID-19 vaccinations for persons who have recovered from previous COVID-19 infection. **Health education video 11:** Importance of early testing. **Health education video 12:** Arrangement for early testing.	Tips 10–12	Reminder of vaccination	
Week 5	**Health education video 13:** Self-protection behaviors. **Health education video 14:** How to perform handwashing properly.	Tips 13–15	Individual consultant	
Week 6	**Health education video 15:** How to wear masks properly. **Health education video 16:** QUIZ: test your COVID-19 knowledge.	Tips 16–18	Reminder of vaccination	
Week 7–8 (Booster session)	Review health education videos 1–6	Tips 19–24	Individual consultant and Reminder of vaccination	
Week 9–10 (Booster session)	Review health education videos 7–12	Tips 25–30	Individual consultant and Reminder of vaccination	
Week 11–12 (Booster session)	Review health education videos 13–16	Tips 31–36	Individual consultant and Reminder of vaccination	**Delivering anti-epidemic package**

* Details of health tips are shown in Supplementary Table S3.

**Table 2:** Baseline characteristics.

Variables ^a^	All Participants (*n* = 50)	Participants with Comirnaty (*n* = 30)	Participants with Sinovac (*n* = 18)	*p* ^b^
Sex				0.614
Male	2 (4.0)	1 (3.3)	1 (5.6)	
Female	48 (96.0)	29 (96.7)	17 (94.4)	
Age (years)	57.1 (9.3)	56.93 (9.34)	58.11 (8.34)	0.663
<60	25 (50.0)	15 (50.0)	9 (50.0)	0.617
≥60	25 (50.0)	15 (50.0)	9 (50.0)	
Chronic diseases				0.762
No	19 (32.0)	11 (36.7)	8 (44.4)	
Yes	31 (68.0)	19 (63.3)	10 (55.6)	
Smoking				0.924
Never	43 (86.0)	27 (90.0)	16 (88.9)	
Ever smoking	4 (8.0)	1 (3.3)	1 (5.6)	
Current smoking	3 (6.0)	2 (6.7)	1 (5.6)	
Drinking				0.592
No	29 (58.0)	18 (60.0)	11 (61.1)	
Yes	21 (42.0)	12 (40.0)	7 (38.9)	
Exercise habit				0.301
No	24 (48.0)	13 (43.3)	10 (55.6)	
Yes	26 (52.0)	17 (56.7)	8 (44.4)	
Employment				0.772
Employed	24 (48.0)	15 (50.0)	8 (44.4)	
Not employed/Retired	26 (52.0)	15 (50.0)	10 (55.6)	
Occupation				0.894
Managers, administrative staff, and professionals	5 (20.8)	3 (20.0)	1 (12.5)	
Clerk	16 (66.7)	10 (66.7)	6 (75.0)	
Service workers, sales and others	3 (12.5)	2 (13.3)	1 (12.5)	
Education				0.744
Primary or below	3 (6.0)	2 (6.7)	1 (5.6)	
Secondary or matriculation	28 (56.0)	18 (60.0)	9 (50.0)	
Tertiary or above	19 (38.0)	10 (33.3)	8 (44.4)	
Marital status				0.680
Single	12 (24.0)	6 (20.0)	5 (27.8)	
Married/Cohabiting	30 (60.0)	19 (63.3)	10 (55.6)	
Divorced/Separated/Widowed	8 (16.0)	5 (16.7)	3 (16.7)	
Living				0.451
With family	42 (84.0)	26 (86.7)	14 (77.8)	
Alone	8 (16.0)	4 (13.3)	4 (13.3)	
Residential area				0.140
Hong Kong Island	7 (14.0)	5 (16.7)	2 (11.1)	
Kowloon	16 (32.0)	12 (40.0)	3 (16.7)	
New Territories	27 (54.0)	13 (43.3)	13 (72.2)	
Income (per month)				0.453
≤10,000 HKD	7 (14.0)	4 (13.3)	3 (16.7)	
10,000–29,999 HKD	14 (28.0)	8 (26.7)	5 (27.8)	
30,000–49,999 HKD	10 (20.0)	8 (26.7)	2 (11.1)	
≥50,000 HKD	6 (12.0)	2 (6.7)	4 (22.2)	
Prefer not to disclose	13 (26.0)	8 (26.7)	4 (22.2)	
Ever taken influenza vaccine	12 (24.0)	5 (16.7)	7 (38.9)	0.101
Took influenza vaccine in the past year	8 (16.0)	4 (13.3)	4 (22.2)	0.451

^a^. Values were reported as mean (SD) or *n* (%), when appropriate. ^b^. *p* values were obtained from chi-squared test and independent *t* test, when appropriate.

**Table 3:** Participants receiving a new dose of COVID-19 vaccine after intervention.

	Participants Receiving New Dose		Raw Vaccination Rate		Modified Vaccination Rate ^a^	
		*n*	Rate	95% CI	Rate	95% CI
1.5th month (*n* = 46)	New vaccine (anyone)	28	60.9%	45.4–74.9%	73.3%	58.1–85.4%
	New 3rd dose	25	62.5%	45.8–77.3%	76.9%	60.7–88.9%
	New 2nd dose	3	75.0%	19.4–99.4%	75.0%	19.4–99.4%
3rd month (*n* = 45)	New vaccine (anyone)	33	73.3%	58.1–85.4%	94.3%	80.8–99.3%
	New 3rd dose	30	76.9%	60.7–88.9%	93.8%	79.2–99.2%
	New 2nd dose	3	75.0%	19.4–99.4%	100.0%	29.2–100.0% ^b^

^a^: The modified vaccination rate was computed by excluding participants infected with COVID-19 during the intervention period. ^b^: One-sided 97.5% CI.

**Table 4:** Behavior of early testing and self-protection ^a^.

Behavior	Baseline	1.5 Months	*p* ^b^	3 Months	*p* ^b^
Early testing—oneself	59.2% (44.2–73.0%)	78.3% (63.7–89.1%)	0.018 *	86.7% (73.2–95.0%)	0.021 *
Early testing—family	57.1% (42.2–71.2%)	78.3% (63.7–89.1%)	0.021 *	84.4% (70.1–93.5%)	0.004 **
Hand washing	9.1 (1.6)	9.4 (1.3)	0.088	9.5 (1.0)	0.037 *
Mask wearing	4.9 (1.3)	5.2 (1.0)	0.042 *	5.3 (1.2)	0.048 *
Social distancing	7.6 (1.3)	8.0 (1.1)	0.017 *	7.5 (1.1)	0.901

^a^: Values were presented as percentage or mean (SD) when appropriate. ^b^: *p* value was obtained from paired *t*-test or McNemar’s test when appropriate. *: *p* < 0.05, **: *p* < 0.01.

## Data Availability

The data that support the findings of this study are available from the first author, Dr. Yao Jie Xie, upon reasonable request.

## Supplementary Materials

The following supporting information can be downloaded at https://www.mdpi.com/article/10.3390/vaccines12090979/s1, Table S1: Assessment results of the phase 1 to phase 4 of the PRECEDE-PROCEED model; Table S2: Decision matrix for each factor in phase 3 of the PRECEDE-PROCEED model; Table S3: Health tips delivered to participants.
