# Supplementary material for: Use of the PRECEDE-PROCEED Model in Piloting Vaccine Promotion and Infection Self-Protection: Intervention Development and Effectiveness Examination
Source: Vaccines (Basel). 2024 Aug 28;12(9):979. doi: 10.3390/vaccines12090979 (PMC11436129; doi:10.3390/vaccines12090979)
Supplement: Supplementary file 1 [file vaccines-12-00979-s001.zip › vaccines-3120257-supplementary.pdf]

Supplementary Table S1: Assessment results of the phase 1 to phase 4 of the PRECEDE-PROCEED model

Phases	Assessment results
**Phase 1 Social assessment**	Evidence from literature review:<br>● A cross-sectional study with 500 Hong Kong residents found that almost 70% of the participants were worried about contracting COVID-19; approximately 40% of participants were bothered by the lack of sufficient surgical masks; all these negative effects of COVID-19 were associated with poorer health-related quality of life across different domains [1].<br>● An online survey amongst 417 Hong Kong Chinese women showed that 32.2%, 42.4%, and 44.9% of participants had negative emotions of stress, anxiety, and depression, which were related to poor quality of life [2].<br>Summary:<br>● This evidence demonstrated that COVID-19 pandemic impacted the quality of life and the well-being of Hong Kong population.
**Phase 2 Epidemiological assessment**	Evidence from literature review:<br>● During wave 1–4 of COVID-19 in Hong Kong, 12,631 residents were infected, corresponding to 1.6 cases per 1000 population [3, *].<br>● The weekly mortality rate of COVID-19 (weekly number of COVID-19 deaths over person-times observed) ranged from 0 to 2.3 per 10,000 person-weeks [3].<br>● Before wave 5, approximately 70% of the population received ≥ 2 doses, but fewer than 10% had received a third dose [3, *].<br>● A cross-sectional study with 1.255 Chinese adults showed that only 39.7% of participants tested for COVID-19 on a voluntary basis [4].<br>● A telephone survey including 31,332 Hong Kong residents observed a declined adherence to voluntary self-protective behaviours during November–December 2020 [5].<br>Summary:<br>● The number of COVID-19 cases, the infection rate and mortality rate of COVID-19 were high in Hong Kong.<br>● The HK population has a relatively low acceptance rate of COVID-19 early testing and vaccination behaviours.<br>● The adherence of self-protective behaviours was insufficient.
**Phase 3 Educational and ecological assessment**	**Predisposing factors:**<br>Evidence from literature review:<br>● A survey among 500 adults showed that only 9.9% to 24%

	participants would like to take a new COVID-19 vaccine dose [6].<br>● A survey among 1008 Hong Kong residents showed that 52.5% participants worried about being infected with COVID-19. And 50.5% participants were confident that they could take measures to protect themselves [7].<br>● A survey among 1201 Hong Kong residents showed nearly one fifth of them were not confident about self-protective behaviours (i.e., face masks, hand soap, and hand sanitiser) [8].<br>Summary:<br>● The beliefs and confidence of COVID-19 vaccine and self-protective behaviours were relatively low<br><br>**Enabling factors:**<br>Evidence from literature review:<br>● A survey among 765 Hong Kong general adults 84.1% of the participants worried about insufficient medical supplies (i.e., face masks, hand sanitiser) in Hong Kong [9].<br>● During the early stage of the COVID-19 outbreak, the Hong Kong Hospital Authority admitted that they underestimated the demand of isolation wards and personal protective equipment [10].<br>Summary:<br>● The availability and accessibility of personal protective equipment were inadequate<br>**Reinforcing factors:**<br>Evidence from literature review:<br>● Health Promotion Branch Centre for Health Protection Department of Health reported that COVID-19 containment measures were difficult to reach to adolescents and young adults [11].<br>● Persons living in public rental housing, and living in an area with low education were associated with longer time to diagnosis in the first wave of infections [12].<br>Summary:<br>● The social supports for COVID-19 vaccine, early-testing, and self-protective behaviours were insufficient
**Phase 4 Administrative and policy assessment and intervention alignment**	Evidence from literature review:<br>● A case report indicated that public sector may fail to convey transparent, comprehensive, and balanced messages about the COVID-19 vaccines, resulting in a public misunderstanding [13].<br>● A modelling study summarised Cantonese COVID-19 fake news and indicated that the fake news weakened public trust toward authorities [14].<br>Summary:

	● Many people did not fully understand the educational materials distributed by the government.<br>● Many people did not fully accept the advice recommended by the government.

* Period for epidemic wave 1-4: 1/1/2020-30/4/2021; Period for epidemic wave 5: 31/12/2021-22/5/2022.

Supplementary Table S2: Decision matrix for each factor in phase 3 of the PRECEDE-PROCEED model

	More Important	Less Important
More Changeable	High Priority for Program Focus:<br>➢ The beliefs and confidence of COVID-19 vaccine<br>➢ The beliefs and confidence of self-protective behaviours	Low Priority Except to Demonstrate Change for Political Purposes:<br>➢ The social supports for early-testing
Less Changeable	Priority for Innovative Program/Evaluation Crucial:<br>➢ The availability of personal protective equipment<br>➢ The accessibility of personal protective equipment<br>➢ The social supports for COVID-19 vaccine<br>➢ The social supports for self-protective behaviours	No Program:<br>NA

Supplementary Table S3: Health tips delivered to participants

Health Tips	Content
Tips 1	Pamphlet of vaccine program in Hong Kong
Tips 2	Dynamic broadcast of "Stay Home Safe" Scheme
Tips 3	The arrangement of vaccine pass initiative
Tips 4	The guidelines for COVID-19 treatment.
Tips 5	Government calls on Covid-19 rapid antigen tests
Tips 6	Introduction of vaccination schemes for high-risk groups
Tips 7	Introduction of vaccine effectiveness of one, two, and three doses of BNT162b2 and CoronaVac against COVID-19 in Hong Kong
Tips 8	Introduction of the Chinese medicine anti-epidemic plans
Tips 9	Introduction of Home hygiene measures
Tips 10	How COVID-19 patients seek medical care in Hong Kong?
Tips 11	Introduction of community vaccination centers open at night and on weekends
Tips 12	Dynamic update of COVID-19 cases in Hong Kong
Tips 13	Introduction of free transport service for COVID-19 patients
Tips 14	Introduction of mobile vaccination stations in Hong Kong.
Tips 15	Launch of COVID-19 "Vaccination Day"
Tips 16	Presentation of latest situation of COVID-19 infection in Hong Kong
Tips 17	Introduction of self-protective measures against COVID-19 in Hong Kong
Tips 18	Dynamic update of vaccination sites in Hong Kong.
Tips 19	Dynamic update of COVID-19 cases in Hong Kong
Tips 20	Government guide about how to get free Rapid Antigen Test kits in Hong Kong?
Tips 21	Fourth dose COVID-19 vaccination arrangements for persons aged 60 or above
Tips 22	Private doctors allow to order two antivirals Paxlovid and Molnupiravir for COVID-19 patients.
Tips 23	Government guide about what should I do when there is a confirmed case in my building?
Tips 24	Government guide about how to eat healthily during COVID-19?
Tips 25	Government guide about how to exercise properly during COVID-19?
Tips 26	Handbook for personal hygiene
Tips 27	Advice on health measures for persons tested positive
Tips 28	Introduction of long COVID
Tips 29	How to choose Rapid Antigen Test kits?
Tips 30	How to maintain healthy lifestyle during COVID-19?
Tips 31	The effectiveness of vaccines in reducing the risk of subsequent long-COVID
Tips 32	How to properly perform hand hygiene?
Tips 33	How to properly use masks?
Tips 34	How to maintain cough manners?
Tips 35	Handbook for household hygiene
Tips 36	How to alleviate COVID-19 related negative emotions?

Reference:

1. Choi EPH, Hui BPH, Wan EYF, Kwok JYY, Tam THL, Wu C. COVID-19 and Health-Related Quality of Life: A Community-Based Online Survey in Hong Kong. Int J Environ Res Public Health. 2021; 18.
2. Hung MSY, Lam SKK, Chan LCK, Liu SPS, Chow MCM. The Psychological and Quality of Life Impacts on Women in Hong Kong during the COVID-19 Pandemic. Int J Environ Res Public Health. 2021; 18.
3. Yang B, Lin Y, Xiong W, Liu C, Gao H, Ho F, et al. Comparison of control and transmission of COVID-19 across epidemic waves in Hong Kong: an observational study. Lancet Reg Health West Pac. 2024; 43:100969.
4. Yan E, Lai DWL, Lee VWP, Ng HKL. Predicting Public Adherence to COVID-19 Preventive Measures: A Cross-Sectional Study in Hong Kong. Int J Environ Res Public Health. 2021; 18.
5. Cowling BJ, Ali ST, Ng TWY, Tsang TK, Li JCM, Fong MW, et al. Impact assessment of non-pharmaceutical interventions against coronavirus disease 2019 and influenza in Hong Kong: an observational study. Lancet Public Health. 2020; 5:e279-e88.
6. Xiao J, Cheung JK, Wu P, Ni MY, Cowling BJ, Liao Q. Temporal changes in factors associated with COVID-19 vaccine hesitancy and uptake among adults in Hong Kong: Serial cross-sectional surveys. Lancet Reg Health West Pac. 2022; 23:100441.
7. Chan EYY, Huang Z, Lo ESK, Hung KKC, Wong ELY, Wong SYS. Sociodemographic Predictors of Health Risk Perception, Attitude and Behavior Practices Associated with Health-Emergency Disaster Risk Management for Biological Hazards: The Case of COVID-19 Pandemic in Hong Kong, SAR China. Int J Environ Res Public Health. 2020; 17.
8. Hsing JC, Ma J, Barrero-Castillero A, Jani SG, Pulendran UP, Lin BJ, et al. Influence of Health Beliefs on Adherence to COVID-19 Preventative Practices: International, Social Media-Based Survey Study. J Med Internet Res. 2021; 23:e23720.
9. Chan EYY, Kim JH, Kwok KO, Huang Z, Hung KKC, Wong ELY, et al. Population Adherence to Infection Control Behaviors during Hong Kong's First and Third COVID-19 Waves: A Serial Cross-Sectional Study. Int J Environ Res Public Health. 2021; 18.
10. Kwan WM, Mok CK, Kwok YT, Lam HW, Chan KH, Law TS, et al. Bundled interventions for consumption management and monitoring of personal protective equipment in COVID-19 pandemic in Hong Kong local hospitals. BMJ Open Qual. 2020; 9.
11. Kong HAoH. Risk communication for COVID-19. 2020; Available from: https://icidportal.ha.org.hk/Home/File?path=/Training%20Calendar/161/Risk%20communication%20for%20COVID-19_preparation%20for%20the%20next%20battle.pdf.
12. Wu Y, Yan X, Zhao S, Wang J, Ran J, Dong D, et al. Association of time to diagnosis with socioeconomic position and geographical accessibility to healthcare among symptomatic COVID-19 patients: A retrospective study in Hong Kong. Health Place. 2020; 66:102465.

13. Zhang X. Examining COVID-19 Vaccination Misinformation and Clarification by the Public Sector in Hong Kong: A Study of Cantonese Covid-19 Fake News Detection on Social Media2021.
14. Ziwei Wang MZ, Yu Chen, Yunya Song, Liang Lan*. A Study of Cantonese Covid-19 Fake News Detection on Social Media. In: Yixin Chen HL, Yicheng Tu, Usama Fayyad, Xingquan Zhu, Xiaohua Tony Hu, Suren Byna, Xiong Liu, Jianping Zhang, Shirui Pan, Vagelis Papalexakis, Jianwu Wang, Alfredo Cuzzocrea, Carlos Ordonez, editor. IEEE International Conference on Big Data 2021; Orlando, Florida, United States2021.
